# Supplementary material for: Evaluating the Utility of a Large Language Model in Answering Common Patients’ Gastrointestinal Health-Related Questions: Are We There Yet?
Source: Diagnostics (Basel). 2023 Jun 2;13(11):1950. doi: 10.3390/diagnostics13111950 (PMC10252924; doi:10.3390/diagnostics13111950)
Supplement: Supplementary file 1 [file diagnostics-13-01950-s001.zip › diagnostics-2332268-supplementary.pdf]

Supplementary File S1: Common patient questions

A. Treatment

1. What is the treatment for Helicobacter pylori?

The treatment for Helicobacter pylori typically involves a combination of antibiotics and medications to reduce stomach acid. This combination is effective in eliminating the bacteria in the majority of cases. The specific medications and dosages will be determined by a healthcare provider based on a person's individual circumstances. It is important to take all of the prescribed medications as directed and to finish the entire course of treatment to ensure the bacteria are eliminated.

2. What is the treatment for perianal fistulizing Crohn’s disease?

Perianal fistulizing Crohn's disease is a serious and chronic condition that requires long-term medical treatment. The goal of treatment for perianal fistulizing Crohn's disease is to reduce inflammation, improve symptoms, and prevent the disease from getting worse.

Treatment for perianal fistulizing Crohn's disease typically includes a combination of medications, such as anti-inflammatory drugs, immune system suppressors, and antibiotics. In some cases, surgery may be necessary to repair or remove damaged tissue.

It's important to work closely with your healthcare provider to find the right treatment plan for you. Treatment for perianal fistulizing Crohn's disease can be complex and may require ongoing monitoring and adjustments to ensure the best possible outcome.

3. What is the treatment for Hepatitis C?

The treatment for hepatitis C depends on the specific strain of the virus that a person has and the severity of their infection. In general, the main treatment for hepatitis C is a combination of antiviral medications that are taken orally. These medications work by blocking the virus from replicating and spreading throughout the body, which can help to reduce the symptoms of the infection and prevent liver damage.

4. What is the treatment for esophageal varices?

Esophageal varices are a complication of liver disease, and treatment typically involves addressing the underlying liver problem. This can include medications to reduce pressure in the blood vessels, as well as procedures to block or redirect blood flow away from the damaged liver. In some cases, a liver

transplant may be necessary. It's important to work closely with a healthcare provider to determine the best course of treatment.

5. **What is the treatment for rectal cancer stage 2?**

The treatment for rectal cancer stage 2 will depend on a variety of factors, including the size and location of the tumor, the person's overall health, and their personal preferences. The most common treatments for rectal cancer stage 2 include surgery, radiation therapy, and chemotherapy. Some people may receive a combination of these treatments. It is important for people with rectal cancer stage 2 to discuss their treatment options with their doctor in order to determine the best course of action for their individual situation.

6. **What is the treatment for Hepatitis B?**

The treatment for hepatitis B depends on the specific case and the stage of the disease. In some cases, no treatment may be necessary and the infection may resolve on its own. In other cases, antiviral medications may be used to help the body clear the virus and prevent complications. In severe cases, hospitalization may be necessary to support the body while it fights the infection. It is important to consult a doctor for proper evaluation and treatment of hepatitis B.

7. **What is the treatment for esophageal cancer stage 3?**

The treatment for esophageal cancer stage 3 will depend on several factors, including the specific type and location of the cancer, as well as the overall health of the patient. In general, treatment for stage 3 esophageal cancer may include a combination of surgery, radiation therapy, and chemotherapy. The specific treatment plan will be determined by the patient's healthcare provider based on their individual needs. It is important for patients with stage 3 esophageal cancer to work closely with their healthcare team to develop a treatment plan that is right for them.

8. **What is the treatment for colon cancer stage 3?**

The treatment for colon cancer stage 3 will depend on a number of factors, including the specific characteristics of the cancer, the patient's overall health, and the preferences of the patient and their medical team. In general, treatment for stage 3 colon cancer may include surgery to remove the cancer, chemotherapy and/or radiation therapy to kill cancer cells, and targeted therapy to block the growth and spread of cancer cells. It's important for patients with stage 3 colon cancer to work closely with their medical team to determine the most appropriate treatment plan for their individual situation.

9. **What is the treatment for small bowel Crohn’s disease?**

The treatment for small bowel Crohn's disease may vary depending on the severity of the condition and the symptoms a person is experiencing. In general, treatment for small bowel Crohn's disease may include medications such as anti-inflammatory drugs, immune system suppressors, and biologic therapies. In some cases, surgery may be necessary to remove the damaged part of the small intestine. It is important for individuals with small bowel Crohn's disease to work closely with their healthcare provider to develop an appropriate treatment plan.

10. What is the treatment for ulcerative colitis?

Ulcerative colitis is a chronic inflammatory condition that affects the large intestine (colon) and rectum. The treatment for ulcerative colitis depends on the severity of the condition and the symptoms a person is experiencing. In most cases, a combination of medications and lifestyle changes is used to manage the condition and reduce symptoms. Some common treatments for ulcerative colitis include:

Aminosalicylates: These medications help to reduce inflammation in the colon and are typically the first line of treatment for mild to moderate cases of ulcerative colitis. Examples of aminosalicylates include sulfasalazine, mesalamine, and balsalazide.

Corticosteroids: These medications are used to reduce inflammation and can be taken orally or rectally. They can be effective in managing symptoms, but they can have serious side effects if used for a long time.

Immunomodulators: These medications work by suppressing the immune system, which can help to reduce inflammation. Examples of immunomodulators include azathioprine, mercaptopurine, and methotrexate.

Biologic therapies: These medications are made from proteins that are produced by living cells. They are used to block specific pathways in the immune system that are involved in causing inflammation. Examples of biologic therapies include infliximab, adalimumab, and golimumab.

In addition to medications, making lifestyle changes can also help to manage the symptoms of ulcerative colitis. Some tips for managing the condition include:

Eating a healthy, balanced diet

Getting regular physical activity

Managing stress

Avoiding triggers that can worsen symptoms, such as certain foods or medications

It's important to work closely with your doctor to determine the best treatment plan for your specific case of ulcerative colitis.

11. **What is the treatment for fatty liver?**

The treatment for fatty liver depends on the underlying cause of the condition. In some cases, making lifestyle changes, such as eating a healthy diet and exercising regularly, can help improve liver function and reverse the effects of fatty liver. In other cases, medication or other medical interventions may be necessary. It's important to consult with a doctor to determine the best course of treatment.

12. **What is the treatment for colon cancer stage 4?**

The treatment for colon cancer stage 4 will depend on several factors, including the location and size of the tumor, the extent of the cancer, and the patient's overall health. In general, treatment for stage 4 colon cancer may include a combination of surgery, chemotherapy, radiation therapy, and targeted therapy. The goal of treatment at this stage is to slow the progression of the cancer and improve the patient's quality of life.

13. **What is the treatment for gastric cancer?**

The treatment for gastric cancer will depend on several factors, including the stage of the cancer and the overall health of the patient. Common treatments for gastric cancer include surgery to remove the cancerous tissue, chemotherapy to kill cancer cells, and radiation therapy to shrink the tumor. In some cases, a combination of these treatments may be used. It's important for patients to discuss their treatment options with their doctor to determine the best plan for their individual situation.

14. **What is the treatment for small bowel lymphoma?**

Treatment for small bowel lymphoma typically involves a combination of chemotherapy and radiation therapy. In some cases, surgery may be recommended to remove the cancerous growth. The specific treatment plan will depend on the stage and type of lymphoma, as well as the overall health and preferences of the individual. It's important to work with a healthcare team that has experience in treating this type of cancer to ensure the best possible outcome.

15. **What is the treatment for radiation proctitis?**

Radiation proctitis is a condition that is caused by radiation therapy to the pelvis. The treatment for this condition will depend on the severity of the symptoms and may include medications to control diarrhea and bleeding, as well as other supportive measures. In some cases, surgery may be necessary to repair damaged tissue. It is important to work with your doctor to determine the best treatment plan for you.

16. **What is the treatment for ischemic colitis?**

Ischemic colitis is a condition that is caused by a decrease in blood flow to the colon. The treatment for this condition will depend on the severity of the symptoms and may include medications to control pain and inflammation, as well as other supportive measures. In some cases, surgery may be necessary to repair damaged tissue. It is important to work with your doctor to determine the best treatment plan for you.

17. **What is the treatment for autoimmune hepatitis?**

Treatment for autoimmune hepatitis typically involves the use of medications to suppress the immune system and reduce inflammation in the liver. The specific medications used and the duration of treatment will depend on the severity of the condition and the response to treatment. In some cases, a liver transplant may be necessary. It's important to work closely with a healthcare team experienced in treating autoimmune hepatitis to develop a treatment plan that is right for you.

18. **What is the treatment for GERD?**

The treatment for GERD, or gastroesophageal reflux disease, can vary depending on the severity of the condition and the individual's symptoms. In most cases, lifestyle changes and over-the-counter medications can help to control the symptoms of GERD. Some common treatment options for GERD include eating smaller, more frequent meals, avoiding foods and drinks that can trigger symptoms, and avoiding lying down immediately after eating. Over-the-counter antacids, such as Tums and Rolaids, can also help to neutralize stomach acid and provide relief from symptoms. In more severe cases, prescription medications such as proton pump inhibitors or H2 receptor blockers may be necessary. In some cases, surgery may be recommended to repair the lower esophageal sphincter and prevent acid reflux. It's important to work with your doctor to determine the best treatment plan for your individual needs.

19. **What is the treatment for achalasia?**

The treatment for achalasia depends on the severity of the condition and the symptoms the patient is experiencing. In some cases, medications such as nitrates, calcium channel blockers, or botulinum toxin (Botox) injections can be used to relax the muscles of the esophagus and make it easier to swallow. In more severe cases, surgery may be necessary to widen the esophagus and allow food to pass through. It's important to work with a doctor to determine the best treatment plan for your specific situation.

20. **What is the treatment for diarrhea?**

The treatment for diarrhea depends on the underlying cause of the condition. In many cases, diarrhea can be treated with over-the-counter medications such as loperamide, which can help to slow down the movement of the intestines and solidify the stool. Drinking plenty of fluids and electrolytes can also help to prevent dehydration, which can be a complication of diarrhea. In some cases, it may be necessary to

take antibiotics to treat an infection that is causing the diarrhea. If the diarrhea is severe or persists for more than a few days, it is important to see a doctor for proper diagnosis and treatment.

21. **What is the treatment for carcinoid in the stomach?**

The treatment for carcinoid tumors in the stomach typically involves surgery to remove the tumor. In some cases, medication may be used to help control the symptoms of the tumor, such as diarrhea and flushing. The specific treatment plan will depend on the individual case and may include a combination of different approaches. It is important to work closely with a healthcare provider to determine the best course of treatment.

22. **What is the treatment for pancreatic cyst?**

Pancreatic cysts are fluid-filled sacs that can develop in the pancreas. The treatment for pancreatic cysts depends on the type and size of the cyst, as well as the symptoms it is causing. In some cases, pancreatic cysts may not need treatment and can be monitored with regular imaging tests. However, if the cyst is causing symptoms or is at risk of rupturing or becoming infected, it may need to be treated with surgery. In some cases, a procedure called cyst drainage may be performed to remove the fluid from the cyst. It's important to consult with a doctor to determine the best treatment plan for your specific situation.

23. **What is the treatment for pancreatic cancer?**

There is no standard treatment for pancreatic cancer, as the appropriate treatment depends on various factors such as the stage and location of the cancer, as well as the overall health of the individual. Treatment options for pancreatic cancer may include surgery, radiation therapy, chemotherapy, targeted therapy, or a combination of these. It is important for individuals with pancreatic cancer to work closely with their healthcare team to determine the most appropriate treatment plan for their specific situation.

24. **What is the treatment for biliary stones?**

The treatment for biliary stones, also known as gallstones, depends on the size and location of the stones, as well as the presence of any symptoms. If the stones are small and do not cause symptoms, they may not require treatment. However, if the stones are large or are causing symptoms such as abdominal pain or jaundice, they may need to be removed. This can be done through a procedure called cholecystectomy, in which the gallbladder is removed surgically. In some cases, the stones can be broken up using a procedure called lithotripsy, in which shock waves are used to break the stones into smaller pieces that can be passed naturally. Your doctor will be able to recommend the best course of treatment for you based on your individual situation.

25. **What is the treatment for pancreatitis?**

The treatment for pancreatitis varies depending on the underlying cause and the severity of the condition. Mild cases of pancreatitis may be treated with medications to control pain and nausea, as well as fluids and electrolytes given through a vein (intravenously) to prevent dehydration. More severe cases may require hospitalization for close monitoring and supportive care. In some cases, pancreatitis can be life-threatening and may require intensive care. Treatment may also include antibiotics to prevent or treat infections, and surgery to remove damaged or infected tissue. It's important to work with a healthcare provider to determine the best treatment plan for your individual needs.

26. **What is the treatment for acute diverticulitis?**

The treatment for acute diverticulitis depends on the severity of the condition. Mild cases can be treated with antibiotics and a liquid diet to give the intestines time to heal. More severe cases may require hospitalization and treatment with intravenous antibiotics. In some cases, surgery may be necessary to remove the infected or inflamed part of the colon. It is important to follow the recommended treatment plan to prevent complications and reduce the risk of future episodes of diverticulitis.

27. **What is the treatment for IBS?**

There is no specific treatment for irritable bowel syndrome (IBS), and the treatment plan may vary depending on the individual's symptoms and their severity. The goals of treatment for IBS are to relieve symptoms, improve quality of life, and prevent complications. Treatment options for IBS may include medication, dietary changes, and lifestyle modifications. Some common medications used to treat IBS include laxatives, antidiarrheal drugs, and medications that alter gut motility or reduce pain and discomfort. Making dietary changes, such as eating a healthy and balanced diet and avoiding trigger foods, can also help manage IBS symptoms. In addition, stress management techniques, such as exercise, yoga, and counseling, may be useful in reducing symptoms of IBS. It is important to work with a healthcare provider to develop an appropriate treatment plan for IBS.

28. **What is the treatment for liver cirrhosis?**

The treatment for liver cirrhosis depends on the underlying cause of the condition and the stage of liver damage. In the early stages of cirrhosis, treatment may involve lifestyle changes and medications to control symptoms and slow the progression of the disease. In advanced stages, a liver transplant may be necessary. Some of the medications that may be used to treat liver cirrhosis include beta blockers to control high blood pressure in the liver, diuretics to reduce fluid accumulation, and medications to control symptoms such as fatigue and itching.

29. **What is the treatment for constipation?**

The treatment for constipation will depend on the underlying cause of the condition. In many cases, simple lifestyle changes, such as increasing fluid and fiber intake, and regular exercise, can help to relieve constipation. In other cases, a doctor may prescribe medication to help relieve the symptoms. It is important to talk to a doctor if you are experiencing chronic constipation, as it can be a sign of an underlying medical condition.

30. What is the treatment for GIST?

The treatment for gastrointestinal stromal tumors (GIST) can vary depending on a number of factors, including the size and location of the tumor, as well as the overall health of the patient. In some cases, surgery may be recommended to remove the tumor. In other cases, medication, such as imatinib, may be used to target and shrink the tumor. In some cases, a combination of surgery and medication may be used. It's important to discuss treatment options with a medical professional who is familiar with your specific case.

31. What is the treatment for clostridium difficile?

The treatment for Clostridium difficile (C. difficile) infection typically involves the use of antibiotics. This bacteria can cause symptoms such as diarrhea, abdominal pain, and fever, and can sometimes lead to serious complications if left untreated. The specific type of antibiotic that is used to treat C. difficile infection will depend on the severity of the infection and the individual patient's medical history. In some cases, oral or intravenous vancomycin may be used, while in others, metronidazole or fidaxomicin may be prescribed. In addition to antibiotics, doctors may also recommend supportive care measures such as rehydration and electrolyte replacement to help manage symptoms. It is important for patients to follow their doctor's instructions and complete the full course of treatment to ensure that the infection is fully eradicated.

32. What is the treatment for colon cancer stage 1?

The treatment for colon cancer stage 1 will depend on the specific circumstances of the individual case. In general, surgery to remove the cancerous growth is the most common treatment for stage 1 colon cancer. In some cases, a combination of surgery and chemotherapy may be recommended to ensure that all of the cancerous cells are destroyed. Other potential treatments for stage 1 colon cancer may include radiation therapy or targeted drug therapy. It is important for individuals with colon cancer to discuss their treatment options with their doctor in order to determine the most appropriate course of action.

33. What is the treatment for melanosis coli?

Melanosis coli is a harmless condition that does not require treatment. It is caused by the accumulation of pigment in the cells of the colon, and it is typically a result of long-term use of laxatives. The best way to treat melanosis coli is to stop using laxatives and allow the pigment to be naturally eliminated from

the body. In some cases, drinking plenty of water and eating a high-fiber diet can help to speed up the process. If you are concerned about melanosis coli or if you have any other symptoms, it is best to speak with a doctor for further advice.

34. What is the treatment for diaphragmatic hernia?

A diaphragmatic hernia is a condition in which the contents of the abdomen, such as the stomach, intestines, or liver, move into the chest cavity through a hole in the diaphragm. The treatment for a diaphragmatic hernia depends on the type and severity of the condition. In some cases, it can be treated with medication, while in others, surgery may be necessary to repair the hole in the diaphragm and return the abdominal organs to their proper position. In severe cases, the patient may need to be placed on a ventilator to help them breathe. It is important for anyone with a diaphragmatic hernia to be treated by a doctor who can determine the appropriate course of treatment.

35. What is the treatment for ascending cholangitis?

Ascending cholangitis is a serious infection of the bile ducts that can be life-threatening if not treated promptly. The main treatment for ascending cholangitis is antibiotics, which are typically given intravenously (through a vein) to ensure they are quickly absorbed into the bloodstream. In addition to antibiotics, treatment may also include drainage of the bile ducts, surgical removal of infected or damaged tissue, and supportive care to manage symptoms and prevent complications. It's important to seek medical treatment as soon as possible if you suspect you may have ascending cholangitis.

36. How to treat gastric ulcer?

There are several treatments for gastric ulcers, including medication and lifestyle changes. The specific treatment will depend on the cause of the ulcer and the severity of the condition. Common treatments for gastric ulcers include:

Antacids: These medications neutralize stomach acid and can provide quick relief from the symptoms of gastric ulcers. Antacids can be purchased over-the-counter or prescribed by a doctor.

Proton pump inhibitors (PPIs): These medications reduce the amount of acid produced by the stomach. PPIs are typically taken once or twice daily and can provide longer-term relief from the symptoms of gastric ulcers.

H-2 receptor blockers: These medications block the action of histamine, a substance that stimulates the production of stomach acid. H-2 receptor blockers are taken once or twice daily and can provide relief from the symptoms of gastric ulcers.

Antibiotics: If a bacterial infection is causing the gastric ulcer, antibiotics may be prescribed to kill the bacteria and prevent the ulcer from worsening.

In addition to medication, making lifestyle changes can help manage and prevent gastric ulcers. These changes may include avoiding foods and drinks that irritate the stomach, quitting smoking, and reducing stress. It is important to discuss the best treatment plan with a healthcare provider.

37. How to treat duodenal ulcer?

Duodenal ulcers can be treated with a combination of medications, lifestyle changes, and, in some cases, surgery. The most common treatment for duodenal ulcers is a class of drugs called proton pump inhibitors (PPIs), which work by reducing the amount of acid produced by the stomach. These drugs are typically taken as a pill once or twice a day, and they can help to relieve symptoms and promote healing of the ulcer. In addition to taking PPIs, it is important to avoid foods and beverages that can irritate the ulcer or cause symptoms to worsen, such as spicy foods, caffeine, and alcohol. Avoiding stress and getting enough rest can also help to promote healing. In some cases, a surgical procedure called a pyloroplasty may be necessary to repair the ulcer and prevent it from recurring. It is important to talk to a doctor about the best treatment plan for your specific situation.

38. How to treat hemorrhoids?

Hemorrhoids are a common condition that can cause discomfort and pain. There are several treatment options available, depending on the severity of the hemorrhoids. Some common treatments include:

Over-the-counter creams and ointments to help soothe the area and reduce symptoms such as swelling and pain.

Sitz baths, which involve sitting in warm water for 10-15 minutes several times a day, to help reduce swelling and irritation.

Stool softeners and other medications to help relieve constipation and make it easier to pass stools.

In more severe cases, a doctor may recommend a procedure to remove the hemorrhoids, such as rubber band ligation or hemorrhoidectomy.

It's important to consult with a doctor if you are experiencing symptoms of hemorrhoids, as they can provide advice on the best treatment options for your specific case.

39. How to treat anal fissure?

Anal fissures can be treated in a number of ways, including changes to your diet, medication, and other self-care measures. One of the most effective ways to treat an anal fissure is to increase the amount of fiber in your diet to soften your stools and reduce the strain on your anus when you have a bowel movement. You can also take over-the-counter pain medication, such as ibuprofen or acetaminophen, to help reduce pain and swelling. In some cases, your doctor may prescribe a topical ointment or cream to help relax the muscles in your anus and promote healing. In severe cases, your doctor may recommend surgery to repair the fissure.

40. How to treat stool incontinence?

There are several treatment options for stool incontinence, also known as fecal incontinence. The treatment approach will depend on the underlying cause of the incontinence. Some potential treatment options include:

Dietary changes: Making changes to your diet, such as increasing fiber and fluid intake, can help to improve bowel function and reduce the risk of incontinence.

Medications: Your doctor may prescribe medications to help improve bowel control or to treat any underlying conditions that may be causing the incontinence.

Bowel training: In some cases, your doctor may recommend a bowel training program to help you improve your control over bowel movements. This may involve setting a regular schedule for bowel movements and gradually increasing the amount of time between bowel movements.

Pelvic floor muscle exercises: Strengthening the muscles of the pelvic floor can help to improve bowel control and reduce the risk of incontinence. Your doctor or a physical therapist can teach you how to do these exercises correctly.

Surgery: In some cases, surgery may be necessary to repair damage to the muscles or nerves of the pelvic floor, or to remove any obstructions in the rectum or anus that may be contributing to the incontinence.

It's important to talk to your doctor if you are experiencing fecal incontinence so that they can help you determine the underlying cause and recommend the best treatment approach for your specific needs.

41. Is there a treatment to celiac disease?

Yes, there is a treatment for celiac disease. The most effective treatment for celiac disease is to follow a strict gluten-free diet. This means avoiding all foods that contain gluten, which is a protein found in wheat, barley, and rye. By eliminating gluten from your diet, you can prevent the immune system from attacking and damaging your small intestine, and allow your gut to heal. While following a strict gluten-free diet can be challenging at first, there are many delicious and nutritious gluten-free options available. Additionally, there are many resources and support groups that can help you learn how to manage your diet and live well with celiac disease.

42. What is leaky gut ?

Leaky gut, also known as increased intestinal permeability, is a condition in which the lining of the small intestine becomes damaged, allowing undigested food particles, toxins, and bacteria to leak through the intestinal wall and enter the bloodstream. This can cause a variety of symptoms and health problems, including digestive issues, food sensitivities, and chronic inflammation. It is not a well-defined medical condition, and more research is needed to understand its causes and effects.

B. Symptoms

1. What causes rectal bleeding?

Rectal bleeding can be caused by a variety of conditions, including hemorrhoids, anal fissures, inflammatory bowel disease (such as Crohn's disease or ulcerative colitis), colorectal cancer, or infection. It is important to speak with a doctor if you experience rectal bleeding, as it can be a sign of a more serious condition.

2. What causes abdominal bloating?

There are several potential causes of abdominal bloating, including:

Eating a large meal or eating too quickly

Swallowing air while eating or drinking

Constipation

Overeating

Irritable bowel syndrome (IBS)

Food sensitivities or intolerances

Hormonal changes

Bacterial overgrowth in the small intestine

Celiac disease

Pregnancy

In many cases, bloating can be relieved by making changes to your diet, such as eating smaller meals more frequently, avoiding foods that cause bloating, and getting regular exercise. In some cases,

bloating may be a sign of a more serious condition, such as a food intolerance or an underlying medical condition, so it's always a good idea to speak with your doctor if you're experiencing bloating.

3. **What causes abdominal distention?**

Abdominal distention, also known as bloating, can be caused by a variety of factors, including constipation, excess gas, lactose intolerance, and inflammatory bowel disease. Eating foods that are high in fiber can help to prevent constipation, which can help to reduce bloating. Drinking plenty of water can also help to prevent constipation and reduce bloating. In some cases, bloating may be a sign of a more serious medical condition, so it is always a good idea to talk to your doctor if you are experiencing persistent bloating.

4. **What causes nausea?**

Nausea can be caused by a variety of things, including certain medications, medical conditions, and infections. It can also be a side effect of chemotherapy or radiation therapy. In some cases, nausea may be caused by a disturbance in the balance of fluids and electrolytes in the body, or by a problem with the digestive system. Emotional stress, anxiety, and motion sickness can also cause nausea. If you are experiencing nausea, it is important to talk to your doctor to determine the cause and get appropriate treatment.

5. **What causes recurrent vomiting?**

There are many possible causes of recurrent vomiting, including viral infections, bacterial infections, food poisoning, motion sickness, and certain medications. Other potential causes include digestive conditions such as gastroenteritis, stomach ulcers, and inflammatory bowel disease. In some cases, recurrent vomiting may be a sign of a more serious underlying medical condition, such as a brain tumor or pancreatitis. It is important to consult a doctor if you are experiencing recurrent vomiting, as they will be able to determine the cause and recommend appropriate treatment.

6. **What causes weight loss?**

There are many possible causes of weight loss, including diet and exercise, illness, and certain medications. In many cases, a combination of factors is responsible for weight loss.

7. **What causes weight gain?**

There are many factors that can cause weight gain, including a person's diet, lifestyle, and medical conditions. Some common causes of weight gain include eating a diet high in calories and not getting enough physical activity. Other factors that can contribute to weight gain include certain medical conditions, such as hypothyroidism and polycystic ovary syndrome (PCOS), as well as certain

medications, such as some antidepressants and steroids. Genetics can also play a role in weight gain. Some people are more prone to gaining weight due to their genes, which can make it more difficult for them to maintain a healthy weight.

8. **What causes loss of appetite?**

There are many possible causes of loss of appetite. Some common causes include illness, infection, stress, anxiety, depression, certain medications, and surgery. Additionally, certain medical conditions, such as cancer, liver disease, and kidney disease, can also cause loss of appetite. In some cases, the cause of loss of appetite may be unknown.

9. **What causes bad taste?**

Bad taste can be caused by a variety of things, including certain medications, infections, and underlying medical conditions. In some cases, it may be due to a problem with the taste buds or a problem with the way the brain processes taste signals. It can also be caused by eating spoiled or contaminated food. If you are experiencing a bad taste in your mouth, it is important to see a doctor to determine the underlying cause.

10. **What causes bad breath smell?**

Bad breath, also known as halitosis, can be caused by a variety of things. In most cases, it is caused by bacteria that live in the mouth and produce gases that have a bad odor. These bacteria can grow on the tongue, teeth, gums, or other areas of the mouth. Eating certain foods, such as onions and garlic, can also cause bad breath. Dry mouth, which can be caused by certain medications or medical conditions, can also contribute to bad breath. Smoking and drinking alcohol can also cause bad breath. In some cases, bad breath can be a sign of a more serious medical condition, so it is always best to speak with a doctor if you are concerned about your breath.

11. **What causes dysphagia?**

Dysphagia is the medical term for difficulty swallowing. There can be many different causes of dysphagia, including physical obstructions in the throat or esophagus, neurological disorders, muscle weakness, and certain medications. In some cases, the exact cause of dysphagia may be unknown. It is important for anyone experiencing difficulty swallowing to see a doctor for a proper diagnosis and treatment.

12. **What causes constipation?**

There are several possible causes of constipation, including not eating enough fiber, not drinking enough fluids, not getting enough exercise, certain medications, and certain medical conditions. Constipation

can also be caused by changes in routine, such as traveling or adjusting to a new diet. In some cases, constipation may be a symptom of a more serious underlying condition, so it is important to speak with a doctor if you are experiencing persistent or severe constipation.

13. What causes diarrhea?

Diarrhea is typically caused by a viral or bacterial infection, although it can also be caused by other factors such as consuming contaminated food or drinks, or as a side effect of certain medications. In some cases, diarrhea may also be a sign of a more serious underlying medical condition. It is important to drink plenty of fluids and get plenty of rest if you have diarrhea, and to see a doctor if it lasts for more than a few days.

14. What causes abdominal pain?

Abdominal pain can be caused by a wide range of conditions, including digestive disorders, infections, and certain medical conditions. Some common causes of abdominal pain include:

Indigestion or heartburn

Constipation or diarrhea

Gas or bloating

Food poisoning or stomach flu

Appendicitis

Kidney stones or gallstones

Hernia

Gastroenteritis

Crohn's disease or ulcerative colitis

Endometriosis or pelvic inflammatory disease

Ovarian cyst or fibroid tumors

If you are experiencing severe or persistent abdominal pain, it is important to see a doctor for a proper diagnosis and treatment.

15. What causes reflux?

Reflux, also known as acid reflux, is caused by the upward movement of stomach acid into the esophagus. This can happen when the lower esophageal sphincter (LES), a muscle that acts as a valve between the esophagus and stomach, becomes weakened or relaxed. This allows stomach acid to flow back up into the esophagus, leading to irritation and discomfort. Certain foods and lifestyle factors, such as eating spicy or fatty foods and eating too much, can also increase the risk of reflux.

16. **What causes jaundice?**

Jaundice is caused by an excess of bilirubin, a yellowish substance, in the blood. This can be due to several factors, such as liver disease, excessive red blood cell breakdown, or a blockage in the bile ducts. In some cases, jaundice may be a sign of a more serious underlying condition, so it is important to see a doctor if you have symptoms of jaundice.

17. **What causes enlarged liver?**

There are several potential causes of an enlarged liver, also known as hepatomegaly. Some common causes include excessive alcohol consumption, hepatitis (inflammation of the liver), and fatty liver disease. Other possible causes include certain medications, toxins, and genetic conditions that affect the liver. It's important to consult with a healthcare provider if you have symptoms of an enlarged liver, as it can be a sign of a serious underlying condition.

18. **What causes fatty liver?**

Fatty liver is caused by the accumulation of excess fat in the liver. This can be due to a variety of reasons, such as excessive alcohol consumption, obesity, and diabetes. In many cases, fatty liver is a result of a combination of factors, such as a poor diet, lack of exercise, and genetic predisposition.

19. **What causes enlarged spleen?**

Enlarged spleen is caused by a range of different conditions, including viral or bacterial infections, liver disease, blood disorders, and other conditions that cause the spleen to become overactive. In some cases, an enlarged spleen may be a sign of a more serious underlying condition, so it's important to consult with a doctor if you think you may have an enlarged spleen.

20. **What causes biliary stones?**

Biliary stones, also known as gallstones, can be caused by a variety of factors. The most common cause is an imbalance in the composition of bile, which can cause the bile to form solid crystals. These crystals can then combine to form stones. Other factors that can increase the risk of developing biliary stones

include obesity, rapid weight loss, and certain medical conditions such as liver disease or diabetes. In some cases, the cause of biliary stones is not known.

21. What causes anemia?

Anemia is a condition in which the body lacks enough healthy red blood cells to carry oxygen to the body's tissues. This can be caused by a number of different factors, including:

A deficiency in iron, folate, or vitamin B12, which are essential nutrients for the production of red blood cells

Loss of blood, either through injury or chronic bleeding from conditions such as ulcers or heavy menstrual periods

The destruction of red blood cells, which can be caused by diseases such as sickle cell anemia or thalassemia

The bone marrow's inability to produce enough red blood cells, which can be due to certain medications, cancer, or other underlying medical conditions

Anemia can also be classified based on the size and shape of the red blood cells, which can help determine the underlying cause. For example, if the red blood cells are smaller than normal, this is called microcytic anemia, and it is often caused by iron deficiency. On the other hand, if the red blood cells are larger than normal, this is called macrocytic anemia, and it is often caused by a deficiency in vitamin B12 or folate.

22. What causes change in bowel habits?

There are many potential causes of changes in bowel habits, including diet, medication, and medical conditions. For example, a diet high in fiber can lead to more frequent and larger bowel movements, while certain medications can cause constipation. Medical conditions such as inflammatory bowel disease, irritable bowel syndrome, and infections can also cause changes in bowel habits. It's important to speak with a healthcare provider if you're experiencing significant changes in your bowel habits, as it could be a sign of a more serious underlying condition.

23. What are the symptoms of celiac disease?

The symptoms of celiac disease can vary greatly from person to person and may include abdominal bloating and pain, diarrhea, constipation, pale and foul-smelling stool, weight loss, fatigue, and malnutrition. Other symptoms may include anemia, bone or joint pain, depression, and irritability. In children, celiac disease may cause failure to thrive, delayed growth, and weight loss. Some people with celiac disease may have no symptoms at all. It is important to speak with a healthcare provider if you think you may have celiac disease.

c. diagnosis

1. what is the meaning of high bilirubin in the blood?

High bilirubin levels in the blood, also known as hyperbilirubinemia, can be a sign of a problem with the liver or bile ducts. Bilirubin is a yellowish substance that is produced when red blood cells break down. Normally, the liver processes and removes bilirubin from the blood, but if there is a problem with the liver or bile ducts, bilirubin can build up in the blood and cause symptoms such as yellowing of the skin and eyes (jaundice). High bilirubin levels can also be a sign of other conditions such as Gilbert's syndrome or liver disease. It's important to speak with a doctor if you have concerns about your bilirubin levels.

2. what is the meaning of high CRP in blood exam?

High levels of CRP (C-reactive protein) in the blood can be an indication of inflammation in the body. Inflammation is the body's response to infection, injury, or other types of damage. In some cases, high levels of CRP can be an early sign of a bacterial infection or other serious health condition. It is important to discuss any abnormal test results with a healthcare provider to determine the cause and appropriate treatment.

3. what is the purpose of calprotectin stool test?

Calprotectin is a protein that is found in the digestive system, and it is a marker for inflammation in the intestines. A calprotectin stool test is used to determine the presence and amount of calprotectin in a person's stool, which can help to diagnose inflammatory bowel disease (IBD) or other conditions that cause inflammation in the intestines. By measuring the amount of calprotectin in a person's stool, a healthcare provider can determine the severity of the inflammation and help to determine the most appropriate treatment plan.

4. what are the normal values of stool calprotectin?

Calprotectin is a protein that is found in high levels in the stool of people with inflammatory bowel disease (IBD), such as Crohn's disease and ulcerative colitis. The normal range for calprotectin levels in the stool is typically less than 50 micrograms per gram (µg/g). However, it's important to note that the normal range may vary depending on the laboratory that is performing the test and the specific method used. It's always best to consult with your doctor to determine the normal range for calprotectin levels in your specific case.

5. What causes low hemoglobin in the blood?

There are several possible causes of low hemoglobin levels in the blood, including nutritional deficiencies (such as a lack of iron, folate, or vitamin B12), chronic illnesses (such as kidney disease or cancer), and blood loss. In some cases, low hemoglobin levels may also be due to the body's inability to produce enough red blood cells, which can be caused by bone marrow disorders, certain medications, or other underlying health conditions. It's important to consult with a healthcare provider to determine the specific cause of low hemoglobin levels in the blood.

6. What causes low iron in the blood?

There are many potential causes of low iron levels in the blood, including dietary deficiency, chronic blood loss, certain medical conditions, and the use of certain medications. For example, if an individual is not consuming enough iron-rich foods, or if their body is unable to absorb iron properly, they may develop iron deficiency. Additionally, conditions such as inflammatory bowel disease, celiac disease, and gastric bypass surgery can interfere with iron absorption and lead to low levels of iron in the blood. Finally, certain medications, such as antacids and proton pump inhibitors, can also cause low iron levels. It's important to speak with a doctor to determine the specific cause of low iron levels in the blood.

7. What is the meaning of SOL in CT?

I'm sorry, but I'm not sure what you're referring to. Could you provide some more context or information about what you're asking?

8. What is the meaning of choledocholithiaisis in US?

Choledocholithiasis is the presence of gallstones in the common bile duct. The common bile duct is a tube that carries bile from the liver and gallbladder to the small intestine. When gallstones become stuck in the common bile duct, they can cause a blockage that prevents bile from flowing properly. This can lead to symptoms such as abdominal pain, jaundice, and nausea. Treatment for choledocholithiasis typically involves removing the gallstones through a procedure called endoscopic retrograde cholangiopancreatography (ERCP) or by performing surgery to remove the gallbladder.

9. What is the meaning of dilated CBD in US?

The term "dilated CBD" is not a commonly used term in the United States. It is possible that it could refer to a condition called "dilated cardiomyopathy," which is a type of heart failure in which the heart's ability to pump blood is reduced because the heart's main pumping chamber (the left ventricle) has become enlarged and weakened. However, without more context it is difficult to say for sure what is being referred to.

10. What are the complications of colonoscopy?

There are several potential complications of colonoscopy, although most people do not experience any problems. Some possible complications include:

Perforation (tearing) of the colon: This is a rare but serious complication that can occur if the colonoscope is inserted too forcefully or if the colon is weakened by disease. Symptoms of a perforation may include abdominal pain, fever, and tenderness.

Bleeding: In rare cases, the colonoscope can cause bleeding in the colon. This can usually be treated with medication or by cauterizing the area with electricity.

Infection: There is a small risk of infection after colonoscopy, although this can usually be treated with antibiotics.

Adverse reactions to sedation: Some people may have an allergic reaction to the sedative medication used during the procedure. Symptoms may include rash, hives, difficulty breathing, or swelling of the face, lips, or tongue.

Incomplete examination: In some cases, the colonoscope may not be able to reach the entire length of the colon. This can be due to a number of factors, including a very large or very small colon, a lot of stool in the colon, or strictures (narrowing) in the colon.

Overall, the risks of colonoscopy are generally considered to be low. It is important to talk to your doctor about the potential risks and benefits of the procedure, and to follow their instructions carefully to reduce the chances of complications.

11. What are the complications of gastroscopy?

Gastroscopy is generally a safe procedure, but like any medical procedure, it carries a risk of complications. Some potential complications of gastroscopy include bleeding, infection, and tissue damage. In rare cases, the gastroscopy tube may cause a tear in the lining of the esophagus or stomach. In very rare cases, the procedure can lead to a collapsed lung or a punctured organ. It is important to discuss the potential risks and complications of gastroscopy with your doctor before the procedure.

12. How is colonoscopy performed?

A colonoscopy is a medical procedure in which a doctor uses a specialized instrument called a colonoscope to examine the inside of the large intestine (colon). The colonoscope is a long, flexible tube that has a small camera and light at one end, which allows the doctor to see the inside of the colon on a monitor. The doctor will gently insert the colonoscope into the rectum and slowly guide it through the colon, taking care to avoid damaging the walls of the intestine. The doctor may use air or water to help open up the colon and get a better view. The procedure typically takes 30 to 60 minutes, and the patient will be sedated during the procedure to make it more comfortable.

13. How is gastroscopy performed?

Gastroscopy is a medical procedure that involves inserting a long, thin, flexible tube with a light and camera at the end (called an endoscope) through the mouth and into the stomach. The doctor performing the procedure will first have the patient lie on their side on a table and will administer a local anesthetic to numb the throat. The endoscope is then carefully passed through the mouth and into the esophagus, stomach, and sometimes the first part of the small intestine. The camera on the endoscope allows the doctor to see inside the digestive tract and look for any abnormalities. The procedure typically takes about 20-30 minutes to complete.

14. What is ERCP?

ERC is a medical procedure that is used to diagnose and treat problems that affect the bile ducts, gallbladder, and pancreas. The procedure is called ERCP, which stands for "endoscopic retrograde cholangiopancreatography." It is performed using an endoscope, which is a long, flexible tube with a light and a camera on the end. The endoscope is inserted through the mouth and into the stomach and small intestine, allowing the doctor to see the inside of the digestive tract. During the procedure, a contrast dye is injected into the bile ducts and pancreatic ducts, and X-rays are taken to create images of these structures. ERCP can be used to diagnose and treat a variety of conditions, such as gallstones, pancreatitis, and cancer of the pancreas.

15. How is ERCP performed?

Endoscopic retrograde cholangiopancreatography (ERCP) is a medical procedure that combines endoscopy and fluoroscopy to diagnose and treat certain conditions of the bile ducts, pancreas, and gallbladder. During the procedure, a thin, flexible tube called an endoscope is passed through the mouth and into the stomach and small intestine. The endoscope has a light and a camera at its tip, which allows the doctor to see the inside of the digestive tract. Once the endoscope is in place, a contrast dye is injected into the bile ducts or pancreatic ducts through the endoscope. This dye allows the doctor to see the ducts more clearly on a special X-ray called a fluoroscope. The doctor may also use special tools to take samples of tissue or remove gallstones through the endoscope. ERCP is typically performed by a specially trained gastroenterologist.

16. what are the complications of ERCP?

Endoscopic retrograde cholangiopancreatography (ERCP) is a procedure that is used to diagnose and treat conditions that affect the bile ducts, pancreas, and gallbladder. It involves the insertion of an endoscope (a thin, flexible tube with a light and camera) into the mouth, down the esophagus, and into the small intestine. The endoscope is then used to guide a catheter (a thin, flexible tube) through the bile ducts and into the pancreas.

There are several potential complications associated with ERCP, including pancreatitis (inflammation of the pancreas), infection, bleeding, and perforation (a hole) in the digestive tract. Other complications may include adverse reactions to the sedative medication used during the procedure, damage to the bile ducts or pancreas, and the formation of scar tissue.

In some cases, complications from ERCP may be severe and require hospitalization or additional treatment. It is important to discuss the risks and potential complications of ERCP with your doctor before undergoing the procedure.

17. What is hiatal hernia?

A hiatal hernia is a type of hernia that occurs when a part of the stomach protrudes through the diaphragm into the chest cavity. The diaphragm is a muscle that separates the chest cavity from the abdomen and helps to keep the stomach in place. In a hiatal hernia, a small part of the stomach pushes up through the opening in the diaphragm, causing a bulge in the chest. This can cause symptoms such as heartburn and chest pain. Treatment for a hiatal hernia may involve lifestyle changes, medications, or surgery, depending on the severity of the condition.

18. What is gastritis?

Gastritis is a condition in which the lining of the stomach becomes inflamed. Symptoms of gastritis can include stomach pain, nausea, vomiting, and indigestion. In most cases, gastritis is caused by infection with the bacterium Helicobacter pylori, but it can also be caused by certain medications, excessive alcohol consumption, or chronic stress. Treatment for gastritis typically involves the use of antibiotics to clear the infection, if present, and medications to reduce stomach acid and protect the lining of the stomach. It is important to treat gastritis to prevent complications such as ulcers and stomach bleeding.

19. What is esophagitis?

Esophagitis is a condition in which the lining of the esophagus becomes inflamed. This can cause a range of symptoms, including difficulty swallowing, chest pain, and heartburn. In some cases, esophagitis can also lead to bleeding or ulcers in the esophagus. The condition can be caused by a variety of factors, such as acid reflux or infection. Treatment for esophagitis may include medications to reduce stomach acid and promote healing, as well as lifestyle changes to help prevent the condition from recurring.

20. what is Barrettes esophagus?

Barrett's esophagus is a condition in which the tissue lining the esophagus (the tube that connects the mouth to the stomach) is replaced by tissue that is similar to the lining of the intestines. This change in the lining of the esophagus is often a result of long-term acid reflux, which can cause the normal cells in the esophagus to be replaced by these abnormal cells. Barrett's esophagus is a serious condition

because it can increase the risk of developing esophageal cancer. Treatment for Barrett's esophagus typically involves medications to control acid reflux and regular endoscopic surveillance to monitor for the development of precancerous changes in the esophageal lining.

21. What is EUS?

EUS is an abbreviation that can refer to several different things, depending on the context. Some possibilities include:

Endoscopic ultrasound (EUS): a medical procedure in which a long, thin tube with a light and a ultrasound probe on the end is inserted through the mouth or rectum to examine the digestive tract and surrounding structures.

European Union agency for space: an independent organization that supports the development of European space programs and policies.

Experimental Union of States: a hypothetical political entity consisting of a group of countries or states that agree to work together and pool their resources for the common good.

It's important to have more context to be able to provide a more specific answer. Can you provide more information about what you are looking for?

22. What are the complications of EUS?

EUS, or endoscopic ultrasound, is a procedure that uses a specialized endoscope equipped with an ultrasound probe to produce images of the digestive tract and adjacent structures. Complications of EUS are rare, but can include bleeding, perforation (a hole in the wall of the digestive tract), and infection. In some cases, EUS may also cause discomfort or pain, allergic reactions to the sedative medication used during the procedure, or damage to the endoscope. It is important to discuss any potential risks and complications with your doctor before undergoing EUS.

23. What is FNA?

I'm sorry, but I'm not sure what you are referring to by "FNA." Could you please provide some more context or information?

24. What is biopsy?

A biopsy is a medical procedure in which a small sample of tissue is taken from the body for examination, typically in order to diagnose a disease. The tissue sample is typically removed using a needle or small surgical instrument and is then examined under a microscope by a pathologist in order to determine the presence or absence of abnormal cells. Biopsies are commonly used to diagnose

cancer and other diseases, and they can help doctors determine the best course of treatment for a patient.

25. What is gastric erosion?

Gastric erosion is a condition in which the lining of the stomach becomes inflamed and worn away. This can cause symptoms such as abdominal pain, nausea, vomiting, and loss of appetite. In severe cases, gastric erosion can lead to bleeding and ulcers in the stomach. Treatment for gastric erosion may include medications to reduce stomach acid and promote healing, as well as lifestyle changes to promote healthy digestion. It is important to see a doctor for proper diagnosis and treatment.

26. what is duodenitis?

Duodenitis is a condition that occurs when the duodenum (the first part of the small intestine) becomes inflamed. This can be caused by a number of different things, such as infection,

27. What is angiodysplasia?

Angiodysplasia is a condition in which there are abnormal or dilated blood vessels in the lining of the digestive tract. This can cause bleeding from the digestive tract and can lead to symptoms such as abdominal pain, vomiting of blood, and black or tarry stools. Angiodysplasia is most commonly found in the colon and is more common in older adults. It is typically treated with medication and/or procedures to seal off or remove the abnormal blood vessels.

28. what is colon polyp?

A colon polyp is a growth that develops on the inner lining of the colon, also known as the large intestine. These growths are typically benign (non-cancerous), but some may have the potential to develop into colon cancer over time. Colon polyps can vary in size, shape, and appearance, and they are typically detected during a colonoscopy, a procedure in which a doctor inserts a flexible tube with a camera attached into the rectum and colon to look for abnormalities. The polyps can then be removed during the colonoscopy to prevent them from becoming cancerous. It is important to have regular screening tests for colon polyps, especially if you have a family history of colon cancer or other risk factors for the disease.

29. what is colitis?

Colitis is a term used to describe inflammation of the colon. It can be caused by a variety of factors, such as infection, inflammatory bowel disease, or other conditions. Symptoms of colitis may include abdominal pain, diarrhea, and blood in the stool. Treatment will depend on the underlying cause of the condition.

30. what is ileitis?

Ileitis is a condition that occurs when the ileum, the lower part of the small intestine, becomes inflamed. Symptoms of ileitis may include abdominal pain, diarrhea, and weight loss. Treatment of ileitis typically involves a combination of medications and dietary changes. In some cases, surgery may be necessary to remove damaged or infected tissue.

31. what is liver hemangioma?

A liver hemangioma is a non-cancerous tumor that develops in the liver. It is made up of a cluster of blood vessels and typically does not cause any symptoms. In some cases, however, a liver hemangioma may cause abdominal pain or discomfort. If you are concerned that you may have a liver hemangioma, it is important to speak with your doctor for a proper diagnosis and treatment plan.

32. what is lipoma?

A lipoma is a benign (non-cancerous) tumor made up of fat cells. It typically appears as a soft, rubbery lump under the skin and can vary in size. Lipomas are usually not painful and are commonly found on the neck, shoulders, back, or arms. They are typically harmless and do not typically require treatment, but they can be removed surgically if they cause discomfort or other symptoms.

33. What is lymphatic hyperplasia?

Lymphatic hyperplasia is a condition in which the lymphatic vessels (part of the immune system) become enlarged or dilated. This can lead to swelling in the affected area. It can be caused by various factors, including infection, inflammation, and other underlying medical conditions. Treatment for lymphatic hyperplasia typically involves addressing the underlying cause and managing symptoms.

34. what is capsule endoscopy?

Capsule endoscopy is a medical procedure that uses a small, swallowable camera to take images of the inside of the body. The camera is contained in a capsule that the patient swallows, and as it moves through the digestive system, it takes pictures that are transmitted to a recorder worn by the patient. The resulting images can be used by doctors to diagnose and evaluate various conditions of the digestive tract.

35. What are the complications of capsule endoscopy?

Capsule endoscopy is a relatively safe and non-invasive procedure. However, like any medical procedure, there are some potential complications that can occur. Some of the possible complications of capsule endoscopy include:

The capsule may get stuck in the digestive tract. This is a rare complication, but it can be serious and may require surgical intervention to remove the capsule.

The capsule may cause irritation or injury to the lining of the digestive tract. This is also a rare complication, but it can cause symptoms such as abdominal pain, nausea, or vomiting.

The capsule may not provide a clear enough view of the digestive tract for the doctor to make an accurate diagnosis. In this case, additional tests may be needed to obtain a better view.

There is a small risk of allergic reaction to the materials used in the capsule, such as the coating or the battery.

Overall, the risks of capsule endoscopy are low, and the procedure is generally well-tolerated by most people. However, it is important to discuss the potential risks and benefits with your doctor before undergoing the procedure.

36. What is MRCP?

MRCP is an abbreviation that can stand for several different things:

Magnetic Resonance Cholangiopancreatography, a type of medical imaging test used to visualize the bile ducts and pancreas

Member of the Royal College of Physicians, a professional organization in the United Kingdom for doctors who specialize in the field of internal medicine

Multiple Response Capture Protocol, a protocol used for polling multiple devices on a computer network

Model-based Reasoning in Critical Care, a research project aimed at developing artificial intelligence systems for use in intensive care units

It's important to clarify which meaning is intended in a given context, as these different meanings can be quite different from one another.

37. What is MRE?

MRE is an abbreviation that can refer to a few different things. One possibility is that it stands for "Meals, Ready-to-Eat," which are pre-packaged meals that are designed to be lightweight and easy to store, and that can be eaten without needing to be cooked. These types of meals are often used by the

military, by disaster relief organizations, and by individuals who need to have ready-to-eat food on hand for emergency situations.

38. what is PCT?

PCT stands for "Patent Cooperation Treaty." It is an international treaty that provides a standardized process for filing a patent application in multiple countries. This can make it easier and more cost-effective for inventors and businesses to protect their inventions on a global scale. PCT applications are filed with the World Intellectual Property Organization (WIPO) and are subject to examination by national or regional patent offices in the countries where protection is sought.

39. Can capsule endoscopy diagnose Crohn’s disease?

Yes, capsule endoscopy can be used to diagnose Crohn's disease. During a capsule endoscopy, a small camera in the form of a pill is swallowed by the patient. The camera takes pictures of the inside of the gastrointestinal tract as it passes through, which can then be used to diagnose conditions like Crohn's disease.

40. what is virtual colonoscopy?

Virtual colonoscopy is a non-invasive medical procedure that uses computed tomography (CT) scans to create detailed images of the inside of the colon. It is used to diagnose and evaluate conditions such as colon cancer, inflammatory bowel disease, and polyps. Unlike traditional colonoscopy, which involves inserting a camera into the rectum to examine the colon, virtual colonoscopy uses a CT scanner to take a series of cross-sectional images of the colon from outside the body. These images are then used to create a 3D model of the colon, which can be examined by a doctor to look for abnormalities.

41. What is ASCA?

I'm sorry, I don't have any information about ASCA. It's possible that you have the wrong name or abbreviation, or that ASCA is a relatively unknown organization or concept. Can you provide any additional context or information about what you are asking?

42. What is TTG?

I'm sorry, but I'm not able to browse the internet or access any information outside of my training data. Without any context, I'm unable to provide a definitive answer to your question. Can you please provide some additional information or context?

43. Is gastroscopy mandatory to diagnose celiac disease?

No, a gastroscopy is not always necessary to diagnose celiac disease. In fact, many people are able to receive a diagnosis based on their symptoms and a blood test that checks for antibodies to gluten. A gastroscopy may be recommended in some cases to confirm the diagnosis or to evaluate the extent of damage to the small intestine. It's always best to discuss your specific situation with your doctor to determine the best course of action for you.

44. What is gluten sensitivity?

Gluten sensitivity, also known as non-celiac gluten sensitivity, is a condition in which a person experiences symptoms similar to those of celiac disease (an autoimmune disorder triggered by the consumption of gluten) but does not have the antibodies associated with celiac disease. Symptoms of gluten sensitivity can include bloating, abdominal pain, diarrhea, and fatigue, and can occur after consuming foods that contain gluten. It is important to note that gluten sensitivity is not the same as celiac disease, and the two conditions should not be confused.

45. How to diagnose celiac disease?

Celiac disease is a digestive disorder that is caused by an immune reaction to gluten, a protein found in wheat, barley, and rye. To diagnose celiac disease, a doctor will typically recommend a blood test to check for specific antibodies that are present in the blood of people with the condition. The doctor may also recommend a biopsy of the small intestine to confirm the diagnosis. This is typically done using a small tube that is inserted through the mouth and into the small intestine, where a small sample of tissue can be taken for analysis. It is important to be on a diet that includes gluten at the time of the biopsy, as this will allow the doctor to see the effects of the gluten on the small intestine.
